# Preoperative computed tomography staging of nonmetastatic colon cancer predicts outcome: implications for clinical trials

**DOI:** 10.1038/sj.bjc.6603646

**Published:** 2007-03-13

**Authors:** N J Smith, N Bees, Y Barbachano, A R Norman, R I Swift, G Brown

**Affiliations:** 1 Department of Colorectal Surgery, Mayday University Hospital, Croydon, CR7 7RE, UK; 2 Department of Radiology, Mayday University Hospital, Croydon, CR7 7RE, UK; 3 Department of Medical Statistics and Computing, Royal Marsden Hospital, Sutton, Surrey, SM2 5PT, UK; 4 Department of Radiology, Royal Marsden Hospital, Sutton, Surrey, SM2 5PT, UK

**Keywords:** colonic neoplasm, neoplasm staging, tomography, X-ray computed, prognosis, survival analysis

## Abstract

Colon cancer patients routinely undergo preoperative computed tomography (CT) scanning, but local staging is thought to be inaccurate. We aimed to determine if clinical outcome could be predicted from radiological features of the primary tumour. Consecutive patients at one hospital undergoing primary resection for colon cancer during 2000–2004 were included. Patients with visible metastases were excluded. Preoperative CT scans were reviewed independently by two radiologists blinded to histological stage and outcome. Images of the primary tumour were evaluated according to conventional TNM criteria and patients were stratified into ‘good’ or ‘poor’ prognosis groups. Comparison was made between prognostic group and actual clinical outcome. Hundred and twenty-six preoperative CT scans were reviewed. T-stage and nodal status was correctly predicted in only 60 and 62%, respectively. However, inter-observer agreement for prognostic group was 79% (*κ*=0.59) and 3-year relapse-free survival was 71 and 43% for the CT-predicted ‘good’ and ‘poor’ groups, respectively (*P*<0.0066). This compared favourably with 75 *vs* 43% for histology-predicted prognostic groups. Computed tomography is a robust method for stratifying patients preoperatively, with similar accuracy to histopathology for predicting outcome. Recognition of poor prognosis tumours preoperatively may permit investigation into the future use of neo-adjuvant therapy in colon cancer.

Colorectal adenocarcinoma is the third commonest cancer in the United Kingdom, with 35 000 new cases discovered every year and 16 000 deaths resulting annually (Scottish Cancer Registry, 2005; Northern Ireland Cancer Registry, 2005; Office for National Statistics, 2005; Welsh Cancer Intelligence and Surveillance Unit, 2006). Approximately 70% of cases involve the colon, the remaining 30% involving the anus and rectum. Knowledge of the extent of the primary tumour at initial diagnosis is critical for proper management of disease as the prognosis for patients diagnosed with colon cancer is directly related to stage at presentation. In the UK, most patients with colon cancer undergo preoperative staging with abdomino-pelvic computed tomography (CT) scanning, but this is carried out in order to diagnose or exclude the presence of synchronous metastases, rather than to evaluate the characteristics of the primary tumour itself.

Primary surgery is accepted to be the only potentially curative treatment for colonic cancer. Unlike rectal cancer, where preoperative neoadjuvant therapy has been used with considerable success, there is currently no role for preoperative therapy in colon cancer. Adjuvant radio- or chemotherapy may be offered post-surgery to patients with advanced-stage tumours, as defined by histological staging criteria.

Previous studies examining the usefulness of preoperative CT for staging of colon cancer have judged its ‘accuracy’ by comparisons with histology (Freeny *et al*, 1986; Balthazar *et al*, 1988; Acunas *et al*, 1990; Hundt *et al*, 1999). Although tumour staging has its origins in the histological description of colorectal cancers (Dukes, 1932), its rationale is in the stratification of patients into prognostic categories. It may be argued, therefore, that the value of preoperative staging should not be judged solely according to its ability to predict histology, but also according to its ability to predict outcome. We are not aware of any previous studies that have examined the correlation between CT-based staging and outcome. Therefore, we have designed a study to examine this.

## 

### Aims

1. To examine whether the radiological features of the primary colonic tumour seen on the preoperative CT scan could be used to predict clinical outcome.
2. To compare preoperative CT-based prognostication with post-operative histology (the current ‘gold’ standard).

## MATERIALS AND METHODS

The medical records of all 312 consecutive patients undergoing resection of a colonic carcinoma (defined as any tumour above the peritoneal reflection) at Mayday Hospital between January 2000 and December 2004 were retrospectively reviewed. Of these, 201 had preoperative CT-staging performed. Any patients with visible metastases were excluded, as were those patients who had undergone any preoperative radiotherapy or chemo-radiotherapy. For each eligible patient, information on the patient's age, sex and tumour site was recorded. Carcinomas were staged pathologically according to the Royal College of Pathologists guidelines (Quirke and Williams, 2000) and used the TNM classification (Sobin and Wittekind, 2002) (Table 1). This information was obtained from the original pathology report located in the patient's medical notes. The X-ray filing libraries were searched, and the original preoperative CT scans were retrieved.

### Review of CT images

**Figure 1:**
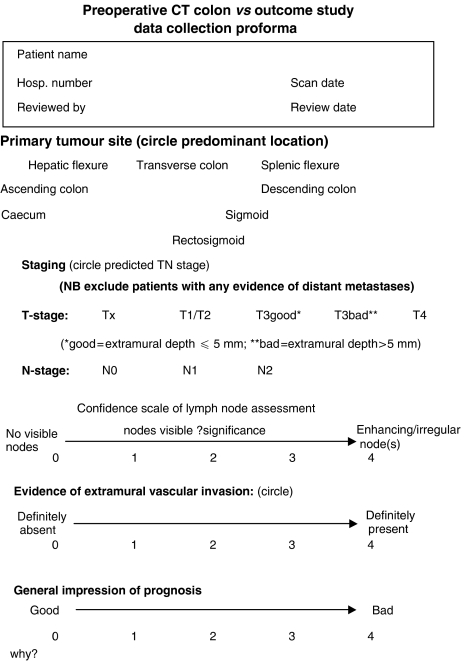
Data collection proforma for CT colon study.

All available preoperative CT films were reviewed independently by two consultant radiologists (NB and GB), both of whom had been involved in previous CT/MRI comparative studies and were experienced with CT-based staging. Each observer was blinded to the final histological stage and clinical outcome. Images of the primary tumour were evaluated using a dedicated proforma (Figure 1) taking into account patterns of local spread derived from previous histological studies as well as conventional TNM assessment (Sobin and Wittekind, 2002).

#### T- and N-stage

The primary tumour was assessed for T-stage. Patients were stratified into ‘good’ or ‘poor’ prognosis groups based on CT-predicted T-stage. Early T3 tumours with predicted extramural invasion up to 5 mm were labelled ‘T3good’ (corresponding to pT3a/pT3b). These together with T1/T2 tumours were stratified into the same favourable prognostic group. More advanced T3 tumours (labelled ‘T3bad’) that appeared to show extramural invasion more than 5 mm beyond the muscle coat (corresponding to pT3c/pT3d) and T4 tumours with suspected involvement or perforation of the visceral peritoneum or direct invasion of an adjacent organ were considered to have a poor prognosis.

In addition, the images were assessed for evidence of lymph node involvement. Lymph nodes were considered to be involved by tumour when they were enlarged or had irregular borders.

#### Overall ‘prognostic score’

A five-point linear scale based on assessment of all the radiological features was used in assigning an overall prognostic score (PS) for each patient.

### Outcome analysis

Prospectively compiled clinical follow-up information was recorded from the medical notes. The date and nature of any disease recurrence (local or distant) was documented and if the patient had died, the date and cause of death. Because the median duration of clinical follow-up was relatively short, relapse-free survival (RFS) was chosen as the outcome measure. Death from any cause and recurrence of colorectal cancer or development of a new colorectal primary tumour were counted as events.

Outcome analysis was performed based on each of the following: histological criteria (Table 2); CT-predicted T-stage; CT-defined PS. For each outcome analysis, patients were allocated into one of two prognostic groups (‘good’ or ‘poor’).

### Statistical analysis

Kaplan–Meier survival curves were used for interpretation of the outcome data. *κ* coefficients were used in the measurement of interobserver agreement.

## RESULTS

### Patient demographics

**Figure 2:**
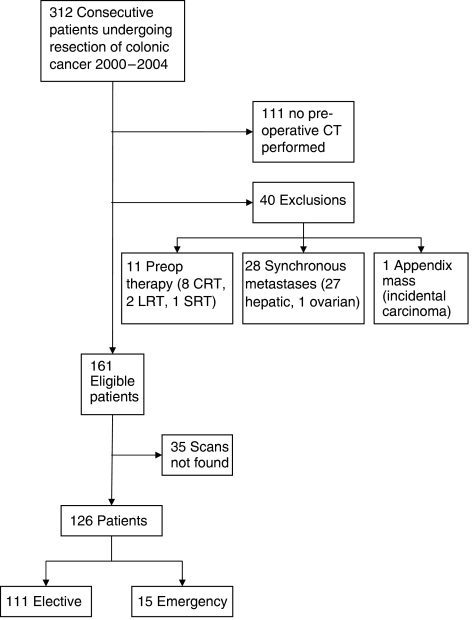
Flowchart for patient selection into study.

During the 5 years between January 2000 and December 2004, 312 patients underwent resection for carcinoma of the colon. Of these, 201 had a preoperative CT scan within a median of 25 days of their operation. Twenty-eight patients had visible metastases on the original CT report, 12 patients had undergone preoperative radiotherapy and one other had an incidental caecal carcinoma resected at laparotomy for an appendix mass. These 40 patients were all excluded. One hundred and twenty six out of 161 (78.3%) scans from 63 men and 63 women were available for review (Figure 2). The median age at operation was 74 years (range 33–89 years).

At the point of survey, 87 patients (69.0%) were still alive, with a median follow-up time since operation of 2.68 years (range=10 months to 6.25 years).

### Accuracy of CT staging

#### T- and N-stage

At histopathological examination, seven (5.6%) of 126 tumours were staged as pT1, 13 (10.3%) as pT2, 67 (53.1%) as pT3 and 39 (31.0%) as pT4.

**Figure 3:**
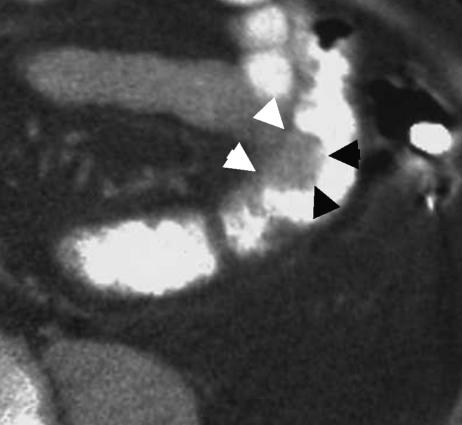
Preoperative CT scan showing a polypoid tumour of the descending colon extending into the lumen (black arrowheads). Tumour does not extend beyond the contour of the muscle coat indicating that this is an early T1/T2 tumour. Pathological staging was pT2.

**Figure 4:**
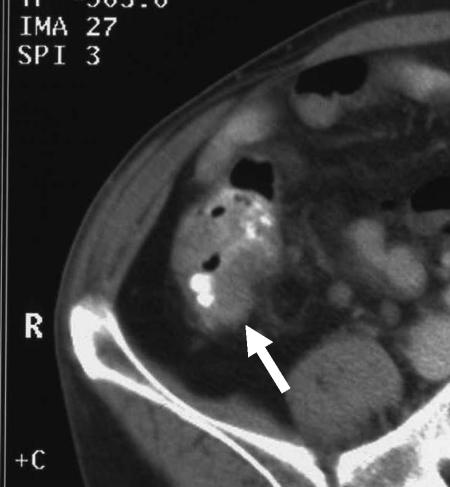
Preoperative CT scan showing a fungating tumour of the ascending colon. The colonic wall is thickened and the posterior contour is irregular owing to tumour projection beyond the non-peritonealised muscle coat. As there is no tumour involvement of the peritonealised surfaces, this is considered a relatively good prognosis T3 tumour. Pathology confirmed a pT3 tumour of the ascending colon.

**Figure 5:**
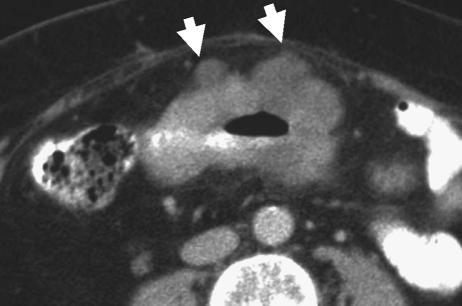
Preoperative CT scan showing a fungating tumour of the transverse colon. The anterior colonic wall is distorted by tumour. As there is minimal pericolic fat and the colon is peritonealised at this location, there is a very high probability that the tumour will be stage T4. Pathological staging was pT4pN1.

**Figure 6:**
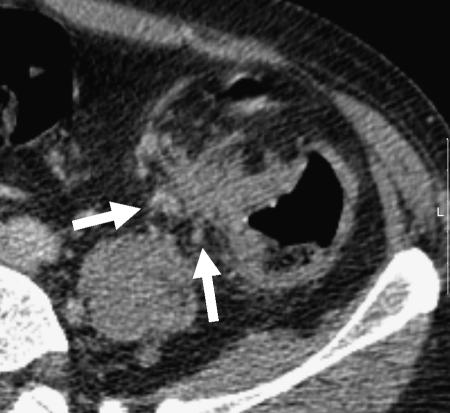
Preoperative CT scan showing a bulky tumour of the descending/sigmoid junction. There is irregular nodular extension medially (arrows) indicating T3 infiltration. This is likely to extend through the medial nonperitonealised, mesenteric surface of the colon. The pathological stage was confirmed as pT3.

The overall accuracy of stage-for-stage prediction of T-stage was 60.3 and 60.8% for observer A and B, respectively (Table 3). For the correct recognition of extramural tumour invasion (stage pT3 or pT4), observer A was 83.3% accurate compared with histology (92.4% sensitivity; 42.1% specificity; positive predictive value (PPV) 89.8%). Observer B achieved 76.2% accuracy with sensitivity, specificity and PPV of 85.9, 61.1 and 92.4%, respectively. Figures 3, 4, 5 and 6 show examples of CT scans evaluated.

Eighty-six (68.3%) of 126 tumours were staged by pathology as pN0, 27 (21.4%) as pN1 and 13 (10.3%) as pN2. The overall accuracy of stage-for-stage prediction of N stage was poor (50.4 and 54.8% for observer A and B, respectively), but for lymph node status (involved or tumour-free), accuracy was slightly better at 61.8 and 62.1% for each observer.

Using the criteria described in Table 2, patients were categorised as having either ‘good’ or ‘poor’ prognosis tumours based on their histological staging. Seventy-one out of 126 (56.3%) patients had tumours categorised histologically as poor prognosis.

#### Prognostic group based on CT T-stage *vs* histology

The best correlation between CT-predicted T-stage prognostic group and histology-predicted group showed an overall accuracy of 71% (sensitivity 73%; specificity 67%), with a PPV (for ‘poor’ prognosis) of 74%. There was 79.2% agreement (*κ*=0.59) between the observers on reporting of advanced T3c/d and T4 tumours (Table 4).

#### Prognostic group based on overall ‘PS’ *vs* histology

The subjective PS (0–4) was also used to assign patients into prognostic categories, with PS=0–2 corresponding to a ‘good’ PS, and PS=3–4 a ‘bad’ score. Correlating PS-based prognostic group with histology-based group, observer A achieved the highest ‘accuracy’ of 69% (sensitivity 73%; specificity 64%), with a PPV (for ‘poor’ prognosis) of 72%. Inter-observer agreement for PS-based prognostic group was 80% (*κ*=0.60).

### Prediction of outcome

Kaplan–Meier survival curves were constructed to illustrate differences in outcome between prognostic groups. As the median length of recorded follow-up was just under 3 years, RFS was chosen as the preferred outcome measure.

#### Histology

**Figure 7:**
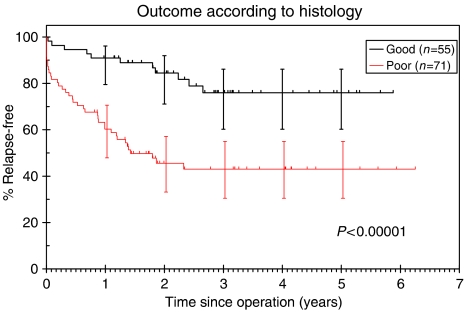
Kaplan–Meier RFS curves according to histological group.

Three-year RFS for patients with ‘good’ prognosis tumours according to histology was 75% (95% confidence interval (CI): 60–86%) *vs* 43% (30–45%) for ‘poor’ prognosis tumours based on histology (*P*<0.00001, Figure 7).

#### Predicted CT T-stage

**Figure 8:**
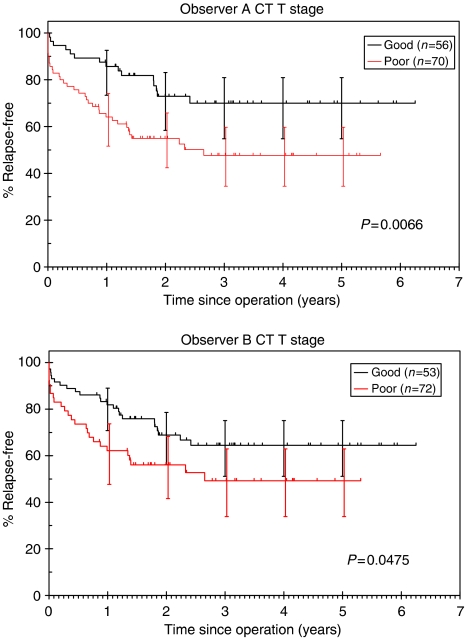
Kaplan–Meier RFS curves according to CT-predicted T-stage.

Three-year RFS for patients with CT-based ‘good’ prognosis tumours was 71% (95% CI: 55–82%) compared with 43% (34–60%) in ‘poor’ tumours for observer A (*P*=0.0066). This compared with 66% (51–75%) *vs* 49% (34–63%) for observer B (*P*=0.0475, Figure 8).

#### Overall CT PS

**Figure 9:**
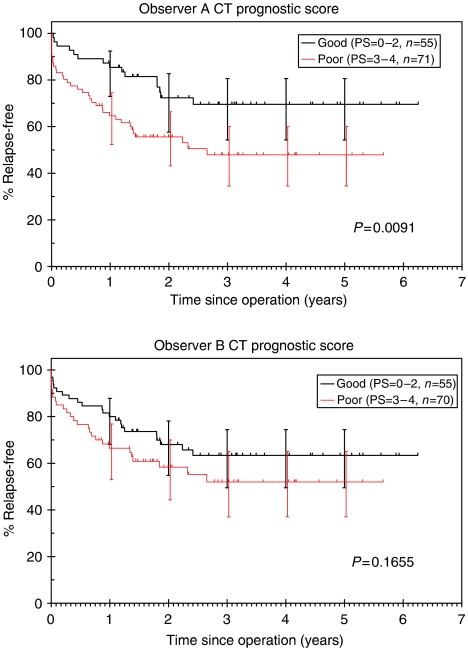
Kaplan–Meier RFS curves according to CT-predicted overall PS.

Three-year RFS for patients with ‘good’ PS (0–2) was 70% (95% CI: 54–81%) compared with 48% (35–60%) for patients with ‘poor’ PS (3–4) for observer A (*P*=0.0091, Figure 9). This compared with 63% (50–74%) *vs* 52% (37–65%) for observer B (*P*=0.1655).

## DISCUSSION

Knowledge of the extent of disease at initial diagnosis is critical for the proper management of patients with colorectal cancer. Several authors have found that preoperative CT provides useful information in up to half and definitely alters clinical management in around 20% of patients with colon cancer (Kerner *et al*, 1993; Barton *et al*, 2002; Mauchley *et al*, 2005) and most often this relates to the detection of liver metastases. In our series, we excluded patients with preoperatively detected distant metastases in order to investigate whether, contrary to conventional wisdom, preoperative CT staging could be useful in predicting outcome in the majority of patients who do not have metastatic disease. No other published series has compared CT-predicted tumour stage with clinical outcome.

We were careful to apply the accepted pathological TNM definitions of T-stage (Table 1) to CT staging. The overall accuracy of T-stage prediction by each of our observers was 60.3 and 60.8% compared with histology, whereas identification of T3/T4 tumours was 85.9–92.4% sensitive, with a PPV of at least 90–92%. These results appear to compare favourably with other studies, which have reported sensitivities of between 55 and 61% and specificities of 67–81% in the detection of ‘serosal’, ‘extramural’ or ‘local (T3–T4) invasion (Freeny *et al*, 1986; Acunas *et al*, 1990; Zerhouni *et al*, 1996). However, this relative lack of definition of depth of invasion makes direct comparison between those studies and our own results difficult. Newer multi-slice/spiral-CT scanners, which are able to collect 1-mm thick slices, within a single breath hold permitting 3D reconstruction, give improved resolution and image quality. Two small series using CT colonography (‘virtual colonoscopy’) have produced good results, with correct T-staging reported in 26 (78.7%) of 33 and 30 (81%) of 37 colorectal tumours (Hundt *et al*, 1999; Laghi *et al*, 2002).

The reported sensitivities for lymph node detection range between 19 and 97% (Freeny *et al*, 1986; Acunas *et al*, 1990; Zerhouni *et al*, 1996; McAndrew and Saba, 1999; Laghi *et al*, 2002). The difficulties of staging lymph nodes based on size criteria are well known – small/normal-sized nodes may have microscopic tumour infiltration, whereas, occasionally, very large nodes may be reactive, rather than malignant. In our series, lymph node status was accurately predicted by each of the two observers in 61.8 and 62.1%, respectively. Consequently, we have not found CT to be particularly useful in the assessment of lymph nodes.

Most published series have emphasised the value of preoperative CT staging to diagnose metastatic disease (Freeny *et al*, 1986; Balthazar *et al*, 1988; Acunas *et al*, 1990; Kerner *et al*, 1993; Thoeni, 1997). Many have concluded that preoperative CT staging is of little value other than to exclude distant metastases because of its limited accuracy, particularly in staging early tumours (Thoeni, 1997). Clearly, no imaging modality that evaluates gross morphology (such as CT) will ever be as ‘accurate’ as the microscope. Nevertheless, although it is undoubtedly true that CT does not ‘accurately’ measure depth of invasion in early-stage tumours, this is not usually of great clinical significance. The clinical outcome for patients with early-stage (T1-2, N0) colonic tumours is usually excellent (5-year overall survival 80–95%). In contrast, patients with locally advanced T4 tumours have a much poorer outcome and some histological studies have also identified extramural invasion greater than 5 mm (T3c/d) as a poor prognostic feature (Merkel *et al*, 2001) and we have recognised this in our categorisation of CT-predicted T-stage. Currently, patients with poor prognostic features on histology may be offered post-operative adjuvant systemic chemotherapy. Identification of patients with locally advanced colonic tumours preoperatively would be of great importance if preoperative neo-adjuvant therapies were to be considered. We have applied the lessons learnt from using MRI to assess the surgical anatomy of the rectum to our CT-based assessment of colonic tumours. Knowledge of the surgical anatomy of the colon and specifically the distribution of peritonealised and nonperitonealised surfaces has enabled us to improve our assessment of the depth of invasion, the presence of T4 disease (peritoneal perforation) and potential resectability.

This study is interesting because it highlights the heterogeneity of actual clinical outcomes for different pathological stages. Pathological criteria are widely accepted as the basis for prognostication (and therefore as an indication for adjuvant therapy) because there is an abundance of evidence that correlates pathology with outcome for populations. However, within a population of patients, some with ‘good’ prognosis tumours will relapse and some with ‘poor’ prognosis tumours will do well, having been cured by surgery alone, receiving no extra benefit from their adjuvant chemotherapy. In our study, the RFS at 3 years for patients with no adverse pathological features was 75 compared with 43% for those with one or more poor prognosis feature (*P*<0.00001). There is to date no other published study that has directly compared preoperative CT staging of the primary tumour with clinical outcome, but our study does appear to show that CT can also discriminate between ‘good’ and ‘poor’ prognosis tumours, with similar ‘accuracy’ to pathology. Not every ‘poor’ tumour relapses and not every ‘good’ tumour remains disease free, but an overall trend is seen in the population and the 3-year RFS based on CT predicted T-stage criteria was 71 *vs* 43% (*P*<0.0066). The fact that the CT-stage may not correlate exactly to the pathological stage is therefore of less importance as the CT stage predicts outcome as well as pathology for the population.

We attempted to produce an overall PS taking into account all visible features (namely T-stage, nodal status and extramural vascular invasion). We found that this was actually less discriminating than CT-predicted T-stage alone, and the prognostic accuracy of T-stage alone was diluted, rather than enhanced by the combination of other features. We believe that the inaccuracy of CT assessment of lymph node status was the major contributory factor, together with the lack of precise objectivity in defining the criteria for the five-point PS, which would limit reproducibility. We therefore do not recommend the use of such a score in its present form.

High sensitivity rates are desirable to enable the potential therapeutic benefit of preoperative treatments to be offered to as many appropriate individuals as possible. However, particularly from the point of view of clinical trials, high PPVs are of even greater importance to avoid giving unnecessary, potentially toxic treatments to patients, who would not otherwise be offered them, even in the usual post-operative setting. Our study has shown high sensitivity (86–92%) and PPVs (90–92%) for the identification of T3 and T4 tumours by preoperative CT. Furthermore, there is reasonable correlation between CT-predicted ‘poor prognosis’ T-stage (i.e. T4 and T3c and d) and poor histological group (PPV=74–75%). However, the most compelling evidence for CT-based prognostication comes from the survival analysis. If CT-predicted T-stage discriminates between good and poor outcome as well as the accepted pathological criteria (which it does in this study), then it seems no more unreasonable to use preoperative CT to guide preoperative treatment, as it does to use pathological criteria to indicate post-operative therapy. The benefits of preoperative therapy in colon cancer, although theoretically persuasive (potential downstaging, greater tolerability), remain unproven. Reliable preoperative staging is required before any pre- *vs* post-operative chemotherapy trial could be set up, and the results of this relatively small study would require further validation in a multi-centre setting. Nonetheless, our results suggest that preoperative CT may be of greater use than simply the exclusion of distant metastases.

In conclusion, we have demonstrated that preoperative CT can predict the clinical outcome for ‘good’ and ‘poor’ prognosis tumours with the same accuracy as histopathology. Our results suggest that it is therefore a robust method for stratifying patients preoperatively, and recognition of poor prognosis tumours preoperatively may permit investigation into the use of neo-adjuvant therapy in colon cancer. This will form the basis of a future clinical trial.

## Figures and Tables

**Table 1:** Definitions of TNM components in the 6th edition of the AJCC and UICC system for staging cancer of the colon and rectum, 2002 (Sobin and Wittekind, 2002)

**Category**	**Description**
TX	The primary tumour cannot be assessed
T0	No evidence of primary tumour
Tis	Carcinoma *in situ* (intraepithelial or intramucosal carcinoma)
T1	Tumour invades into the submucosa
T2	Tumour invades into the muscularis propria
T3	Tumour invades through the muscularis propria into the subserosa, or into nonperitonealised pericolic or perirectal tissues
	
*Optional subdivision of T3*	
T3a	Minimal invasion: <1 mm beyond the border of the muscularis propria
T3b	Slight invasion: 1–5 mm beyond the border of the muscularis propria
T3c	Moderate invasion: >5–15 mm beyond the border of the muscularis propria
T3d	Extensive invasion: >15 mm beyond the border of the muscularis propria
T4	Tumour directly invades into other organs or structures (T4a) or perforates the visceral peritoneum (T4b)
	
NX	Regional lymph nodes cannot be assessed
N0	No regional lymph node metastases
N1	Metastatic tumour in 1–3 pericolic or perirectal lymph nodes
N2	Metastatic tumour in four or more pericolic or perirectal lymph nodes
	
MX	The presence of distant metastasis cannot be assessed
M0	No distant metastasis
M1	Distant metastasis present

**Table 2:** Prognostic categorisation of primary tumour according to histology

**Histological variable**	**‘Good’ prognosis**	**‘Poor’ prognosis**
T-stage	T1, T2 or T3	T4
N-stage	N0	N1 or N2
EMVI	Absent	Present
Distant metastases^a^	Absent	Present
(e.g., peritoneal seedlings)		

a The presence of visible metastases was an exclusion criterion for the study. In a very few cases; however, peritoneal seedlings were only identified at operation.

**Table 3:** CT-predicted T stage for each observer *vs* histology T stage

	**Observer A CT-predicted T-stage**						**Observer B CT-predicted T stage**					
**Histology**	**T1/T2**	**T3good**	**T3bad**	**T4**	**Tx**	**Total**	**T1/T2**	**T3good**	**T3bad**	**T4**	**Tx**	**Total**
T1	4	2			1	7	5				2	7
T2	4	6	2	1		13	6	1	6			13
T3	7	26	18	15	1	67	12	14	26	9	6	67
T4	1	4	10	24		39	2	4	7	25		38
Total	16	38	30	40	2	126	25	19	39	34	8	125

*Observer A*:

Stage-for-stage accuracy=60.3%.

Extramural invasion (stage T3/T4):

Overall accuracy=83.3% (sensitivity=92.4%; specificity=42.1%).

Positive predictive value=89.8%; negative predictive value=50.0%.

*Observer B*:

Stage-for-stage accuracy=60.8%.

Extramural invasion (stage t3/t4):

Overall accuracy=76.2% (sensitivity=85.9%; specificity=61.1%).

Positive predictive value=92.4%; negative predictive value=44.0%.

**Table 4:** Agreement of CT-predicted good/poor prognosis T stage (Observer A *vs* observer B)

	**Observer A**	
**Observer B**	**T1/2, T3good**	**T3bad, T4**
T1/2, T3good	48	21
T3bad, T4	5	51

Inter-observer agreement=79% (*κ*=0.59).
